# Menthol-induced sedation and hemodynamic safety limits in Nile tilapia: defining a therapeutic window

**DOI:** 10.1007/s10695-026-01681-5

**Published:** 2026-04-13

**Authors:** Clarissa Araujo da Paz, Daniella Bastos de Araujo, Axell Lins, Luciana Eiró-Quirino, Thaysa de Sousa Reis, Júlia Santos da Silva, Luciana Esquerdo Cerquira, Luana Vasconcelos de Souza, Lucas Lima da Rocha, Nilton Akio Muto, Moisés Hamoy

**Affiliations:** 1 Natural Products Pharmacology and Toxicology Laboratory, Institute of Biological Sciences, Federal University of Pará, Belém, R. Augusto Corrêa, Pará, 01, 66075-110 Brazil; 2 Centre for Valorization of Amazonian Bioactive Compounds - Institute of Biological Sciences, Federal University of Pará, Belém, Pará, 66075-110 Brazil

**Keywords:** Menthol, Sedation, Therapeutic window, *Oreochromis niloticus*, Cardiac toxicity, GABA_A_ receptor

## Abstract

Menthol, a natural monoterpene extracted from *Mentha* species, has gained attention as an alternative sedative and anesthetic in aquaculture. However, its physiological safety range and potential sublethal toxicity remain insufficiently defined. This study investigated the cardiac and behavioral effects of menthol sedation in *Oreochromis niloticus*, aiming to identify a therapeutic window and elucidate its underlying mechanisms of action. Juvenile fish were exposed to graded concentrations of menthol (100–250 mg/L) to evaluate induction and recovery times, cardiac rhythm stability, and electrocardiographic parameters, including heart rate, QRS amplitude, and PQ and QT intervals. Additional assays with the GABA_A_ receptor antagonist flumazenil (1 mg kg⁻^1^, i.p.) were performed to determine neurophysiological mediation. Menthol induced a concentration-dependent decrease in heart rate and prolongation of ventricular conduction intervals, characterizing sinus bradycardia without arrhythmia. Concentrations between 100 and 200 mg/L provided effective sedation and rapid recovery while maintaining hemodynamic stability, defining a safe therapeutic window. Above 200 mg/L, pronounced bradycardia and delayed recovery were observed, indicating sublethal cardiac depression. Flumazenil significantly prolonged anesthesia induction, confirming GABA_A_-mediated mechanisms in menthol sedation. These findings establish the physiological safety limits for menthol use in Nile tilapia and highlight its ecotoxicological significance, as excessive or environmental exposure may impair cardiac homeostasis in aquatic organisms. The results contribute to defining safe anesthetic practices and to understanding the toxicodynamic implications of natural sedatives in aquatic ecosystems.

**Graphical Abstract:**

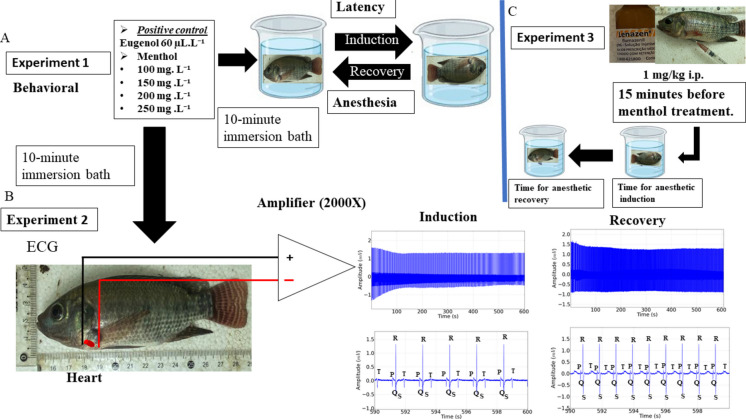

## Introduction

Nile tilapia (*Oreochromis niloticus*) is one of the most important fish species in Brazilian fish farming, accounting for 54% of the country’s total aquaculture production (Valenti et al. 2021). On the international stage, Nile tilapia production has grown exponentially in recent years and the species has become the third most cultivated fish in the world, with global production reaching approximately 4407.2 tons in 2020 (FAO 2022). Therefore, given the importance of this species, it is necessary to find alternatives focused on improving the quality of captive fish production (Aydın & Barbas 2020; Dawood et al. 2021). Improving the welfare of fish during handling in fish farming environments helps to maintain the health of farmed animals. For this to be possible, some procedures are necessary, such as containment through anesthesia. The use of these anesthetic agents, mainly natural, helps to reduce fish mortality by reducing the possibility of injuries and loss of scales during handling and improves the quality of fish meat due to the quality of animal welfare (Boison & Turnipseed 2015; Ross & Ross 2009). The most commonly used anesthetics for Nile tilapia (Oreochromis niloticus), for handling and transport, include eugenol (clove oil), Tricaine methanesulfonate (MS-222), Benzocaine and 2-phenoxyethanol (Rairat et al. 2021).

Menthol is extracted from plants of the genus Mentha, and its crystallized form is produced by the formation of mint oil containing more than 80% menthol; after cooling, menthol is crystallized from the oil (Cohen et al. 2020), being able to promote many effects, such as calming, analgesic, antibacterial, antitumor, improves memory, and has a potential effect on neuroinflammation (Du et al. 2020). Menthol can increase the resistance of Nile tilapia to stressful impacts in captive environments, without reducing growth, increasing antioxidant capacity and immune performance, and it is possible to obtain its complete residual elimination within 48 h (Botrel et al. 2017; Zhang et al. 2008).

The use of natural anesthetics has been evaluated for their effects on the physiology and biochemistry of different fish species, so that safe concentrations can be established (Aydın & Barbas 2020). Menthol is also used therapeutically for pain control, which makes its mechanism of action more complex (Pergolizzi et al. 2018; Xu et al. 2020). Many studies indicate that the mechanism of action of menthol is related to the activation of the GABA_A_ receptor (Zhang et al. 2008; Tani et al. 2010; Lau et al. 2014). Parallel to this, flumazenil is an antagonist of the sedative effects resulting from benzodiazepine overdose, acting on the GABA_A_ receptor, and can replace benzodiazepines by competitive pharmacological antagonism, mainly avoiding respiratory depression, causing rapid recovery in cases of anesthesia caused by benzodiazepines (Penninga et al. 2016).

In this sense, the study aims to evaluate the cardiac safety of juvenile tilapia subjected to menthol, as well as evaluate the underlying mechanisms of action related to the activation of GABA_A_ receptors through blockade with flumazenil in vivo in tilapia.

## Materials and methods

### Experimental animals

A total of 162 juvenile Nile tilapia (*O. niloticus*) were used in the experiments. The animals were kept in five 250-L aquariums in the Experimental Bioterium of the Laboratory of Pharmacology and Toxicology of Natural Products—ICB—Federal University of Pará—UFPA and were randomly selected to compose the experimental groups. The animals were kept in an environment with a temperature (25 to 27 °C) and photoperiod (12/12 h) (light/dark). They were fed commercial feed (32% protein). The aquariums were siphoned to remove leftover feed and feces, with water renewal of 30% of the volume, with water from the same source. The animals were acclimatized for 15 days, during which water quality variables, such as water temperature (°C), hydrogen ion potential (pH) and dissolved oxygen (DO), were monitored and maintained as follows: Temperature 26.11 ± 1.054 °C; pH 7.7 ± 0.25; DO > 5.3 ± 0.02 mg/L. All procedures were approved by the ethics committee (CEUA/UFPA N 1724310322). All experiments were performed using the ARRIVE checklist.

### Experimental design

#### Experiment 1: Behavior observed during exposure to menthol

Alcohol was used as a solvent for menthol, which, due to its chemical properties, does not dissolve in water. Therefore, to assess the possible interference of alcohol, the evaluations were also analyzed for the vehicle group.

The positive control group used eugenol from Laboratório Biodinâmica at 100%, ANVISA registration number: 10298550063.

Tilapia juveniles (28.25 ± 4.5 g) were randomly distributed into the following treatments: (a) control group; (b) vehicle control group (2.5 mL of 70% alcohol per liter of water); (c) Positive control group (treated with 64 mg/L of eugenol), (eugenol density: 1.06 g/cm^3^); (d) treated with menthol 100 mg/L; (d) 150 mg/L; (e) 200 mg L; and (f) 250 mg/L, to evaluate the latency time for loss of postural reflex during the immersion bath. Subsequently, the fish were transferred to water without menthol, where the latency time for recovery of the postural reflex was recorded. The fish were considered recovered when the postural reflex was maintained for at least 15 s (*n* = 9/treatment), totaling 63 animals for experiment 1.

#### Experiment 2: Electrocardiographic evaluation

Tilapia were randomly distributed into the following treatments: (a) control and fish subjected to immersion baths in water without menthol; (b) control vehicle (70% ethanol) 2.5 mL/L of water; (c) positive control group (treated with eugenol 64 mg/L); (d) menthol at a concentration of 100 mg/L; (e) 150 mg/L; (f) 200 mg/L; (g) 250 mg/L. Recordings were made during exposure and recovery at the indicated concentrations. Each recording lasted 10 min and the averages were analyzed between 590 and 600 s, that is, the final ten seconds of exposure to menthol, following the methodology of da Paz et al. (2024). For each group, *n* = 9/treatment were used, totaling 63 animals for experiment 2.

The electrodes were inserted into the ventral part of the opercular opening. The animals were not anesthetized at the time of electrode insertion; however, local anesthesia was performed with 2% lidocaine impregnated in cotton, which was previously placed at the electrode insertion site.

#### Acquisition of electrocardiographic (ECG) recordings

To record tilapia cardiac activity, electrodes were constructed on separate 950 silver rods measuring 0.3 mm in diameter and 2 cm in length. The reference electrode was fixed according to the cardiac vector in derivation D1, positioned ventrally at 0.2 mm from the end of the opercular cavity (left) as the reference electrode, while the recording electrode was fixed at 0.2 mm from the end of the right opercular cavity. The electrodes were then connected to a high-impedance amplifier. Analysis of the recorded graphoelements allowed the evaluation of heart rate in beats per minute (bpm), amplitude of the QRS complex (mV), duration of the QRS complex (ms), PQ (ms), RR (ms), and QT (ms) intervals (Hamoy et al. 2023; da Paz et al. 2024; Reis et al. 2024).

The electrodes were connected to a digital data acquisition system using a high-impedance input differential amplifier (Grass Technologies, Model P511), set with 0.3 and 300 Hz filtering, with 5000 × amplification and monitored by an oscilloscope (Protek, Model 6510). Recordings were digitized continuously at a rate of 1 kHz using a data acquisition board (National Instruments, Austin, TX). The acquired signals were analyzed using a tool built in the Python programming language version 2.7. The Numpy and Scipy libraries were used for mathematical processing and the Matplolib library was used to produce histograms (Hamoy et al. 2023).

#### Experiment 3: Investigation of the mechanisms of menthol anesthetic action

For the experiment, flumazenil (Cristália) (presentation: 0.1 mg/mL), Butantã-São Paulo, was initially applied at a dose of 1 mg/kg i.p. (ultrafine 13 × 0.3 mm insulin needles were used 1 cm posterior to the pectoral fin, without prior anesthesia of the animals), and 15 min after application (so that flumazenil absorption can occur), the animals were placed in an immersion bath with different concentrations of menthol (Indústria Química Anastácio), Sorocaba-São Paulo, taking into account the latency for the loss of the postural reflex during induction and the recovery of the postural reflex after the menthol immersion bath. The animals used in the behavioral study served as control groups for the flumazenil test, with 4 groups of 9 animals each, totaling 36 animals: (a) Flumazenil (1 mg/kg i.p.) associated with 100 mg/L of menthol in an immersion bath, (b) Flumazenil (1 mg/kg i.p.) associated with 150 mg/L of menthol, (c) Flumazenil (1 mg/kg i.p.) associated with 200 mg/L of menthol, and (d) Flumazenil (1 mg/kg i.p.) associated with 250 mg/L of menthol.

### Statistical analysis

**Fig. 1:**
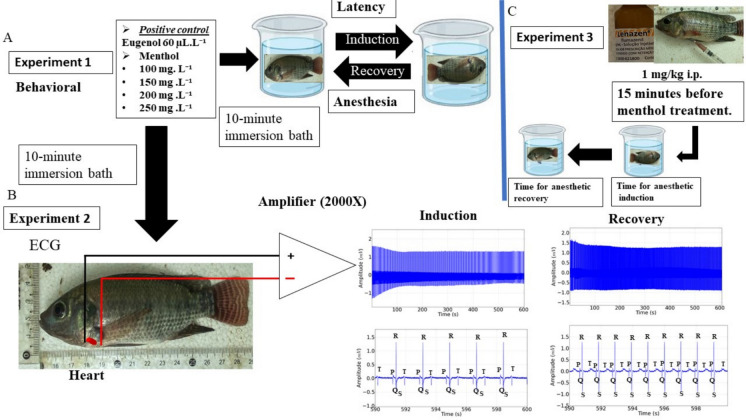
Schematic summary of the experimental design and main findings regarding the concentration-dependent sedative and cardiac effects of menthol in Nile tilapia

After verifying, the assumptions of normality and homogeneity of variance were met using the Kolmogorov–Smirnov and Levene tests, respectively, by the Python program using the SciPy library. Comparisons were made between the mean power of the recordings using one-way ANOVA, followed by Tukey’s post hoc test. GraphPad Prism® 8 software was used for the analyses and values of *p < 0.05, **p < 0.01, ***p < 0.001 were considered statistically significant in all cases (Fig. 1).

## Results

**Fig. 2:**
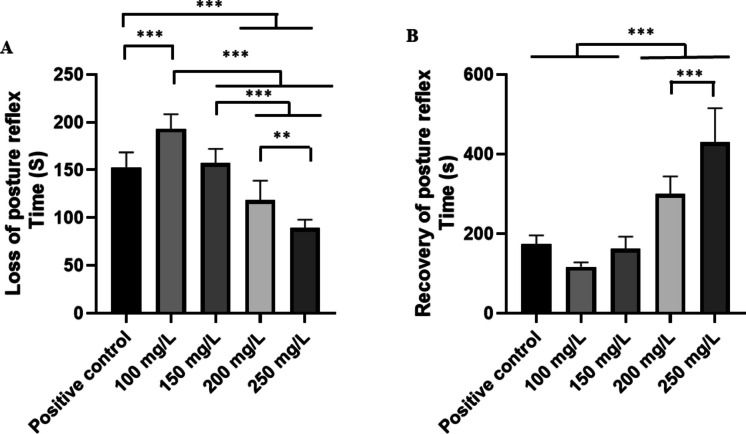
Mean latencies (s) for loss of postural reflex in tilapia during immersion baths with different concentrations of menthol (**A**); recovery of postural reflex after contact with different concentrations of menthol (**B**) (ANOVA followed by Tukey’s test; **p < 0.01 and ***p < 0.001)

Behavioral analysis showed that menthol induced a concentration-dependent loss of postural reflex, and the higher the concentration, the lower the latency. Fish exposed to 100 mg/L of menthol presented loss of posture in 193.1 ± 15.14 s, with longer latency when compared to the other groups: group treated with 150 mg/L (156.9 ± 15.27 s), treated with 200 mg/L of menthol (118.3 ± 20.59 s) and 250 mg/L (89.22 ± 20.59 s). The positive control group was like the group treated with 150 mg/L menthol (*p* = 0.981) (Fig. 2A).

The mean latency for recovery of the postural reflex was lower at lower concentrations. Thus, the group exposed to 100 mg/L of menthol presented similar latency to the group treated with 150 mg/L (*p* = 0.246) and the positive control group (*p* = 0.988); however, the recovery time for the fish exposed to 200 mg/L presented a mean recovery of 299.7 ± 44.29 s, which was greater than the previous groups. The fish treated with 250 mg/L (429.8 ± 85.56 s) was greater than the other groups (Fig. 2B).

**Fig. 3:**
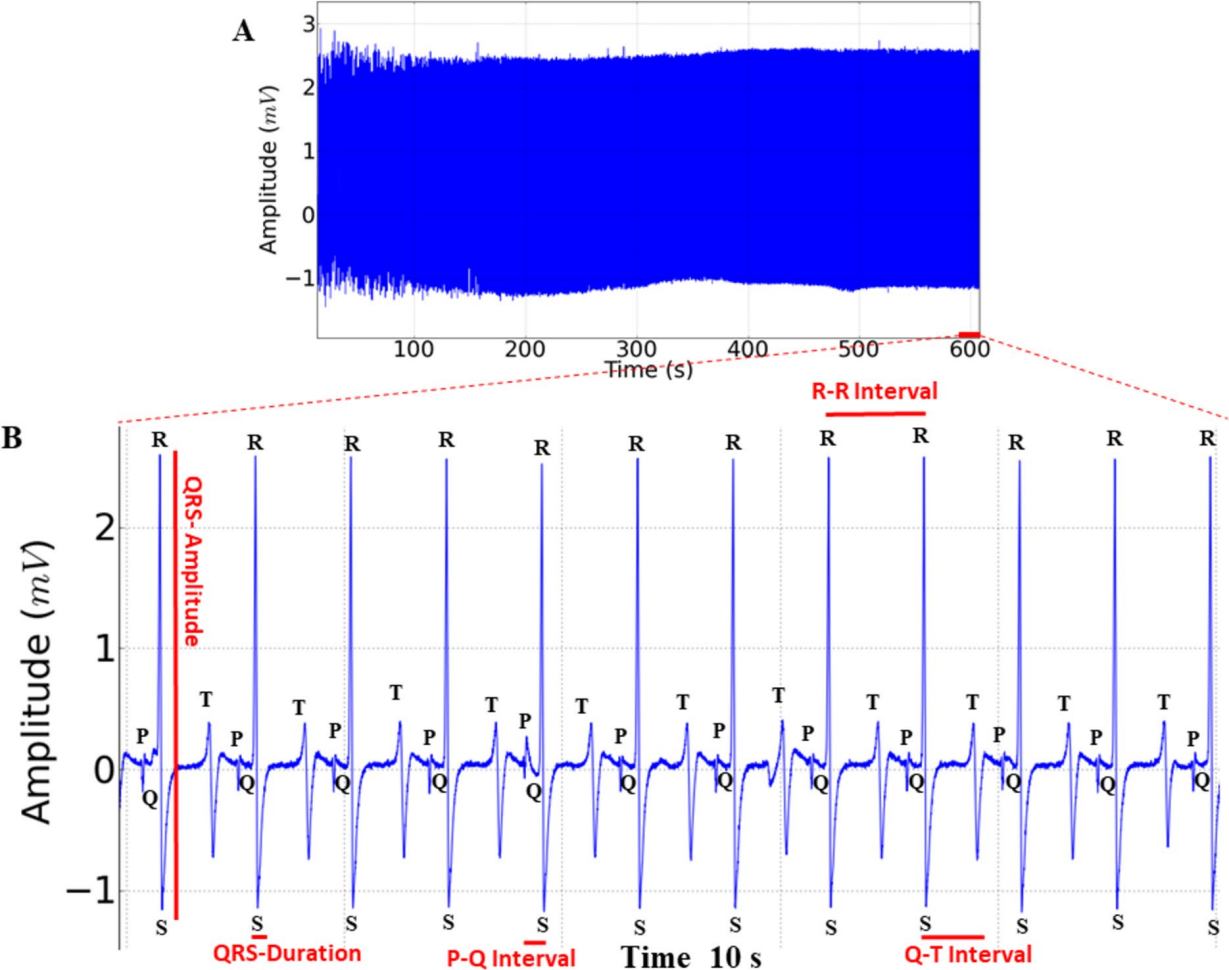
Cardiac activity in juvenile tilapia (**A**) and amplification of the 10-s recording showing the RR, PQ, and QT intervals (ms), QRS complex duration (ms), and QRS complex amplitude (mV), indicated in red (**B**)

Cardiac activity in the controls showed sinus rhythm, with the presence of all cardiac deflagrations in a 10-min recording (Fig. 3A). The mean frequency of 76.89 ± 4.59 bpm, in amplitudes averaging 1.67 ± 0.33 mV, was observed in amplification of the final 10 s of the recording (590–600 s); all the cardiac graphoelements can be identified as atrial activity represented by P waves, ventricular activity by QRS complexes, and ventricular repolarization by T waves (Fig. 3B).

**Fig. 4:**
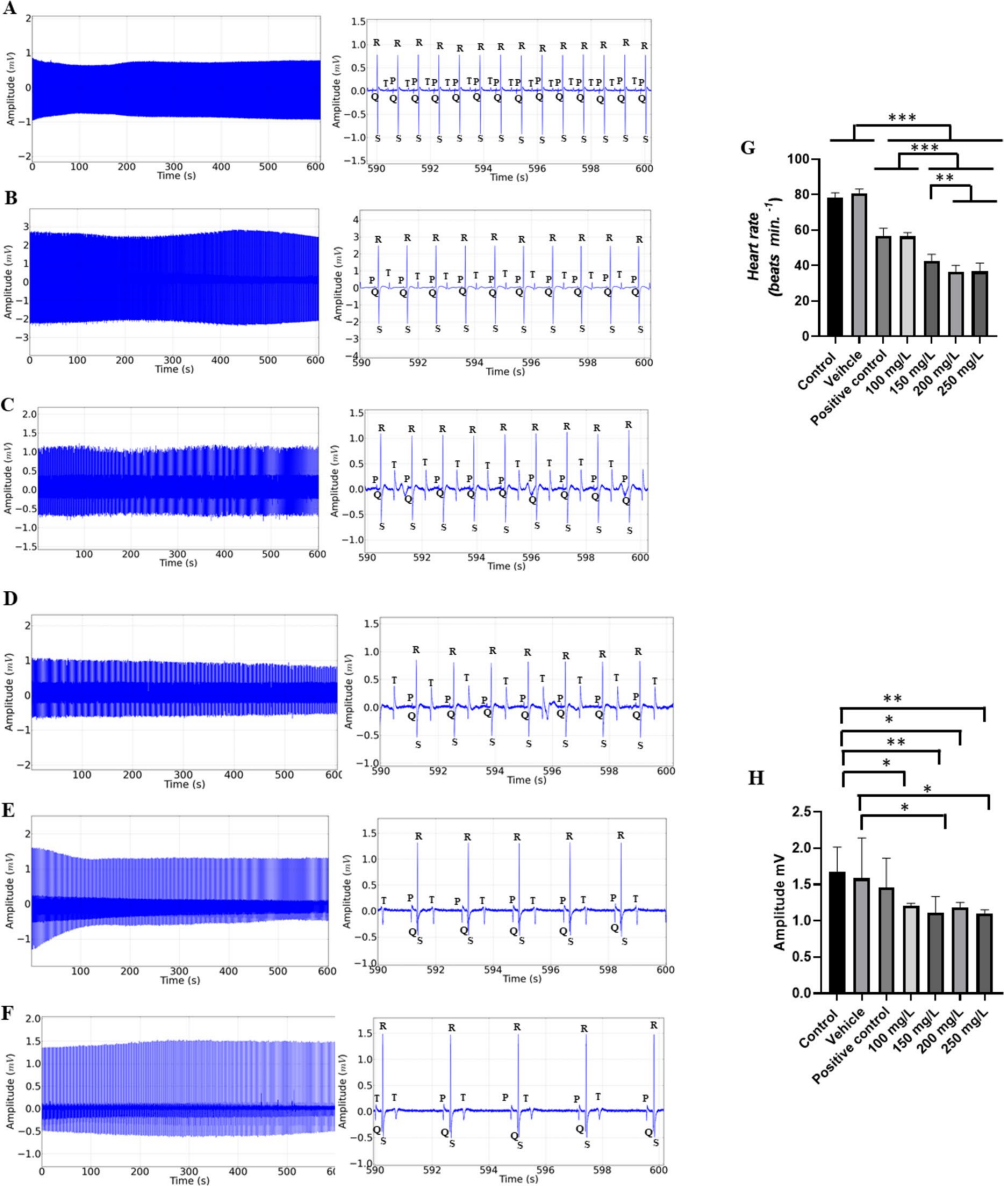

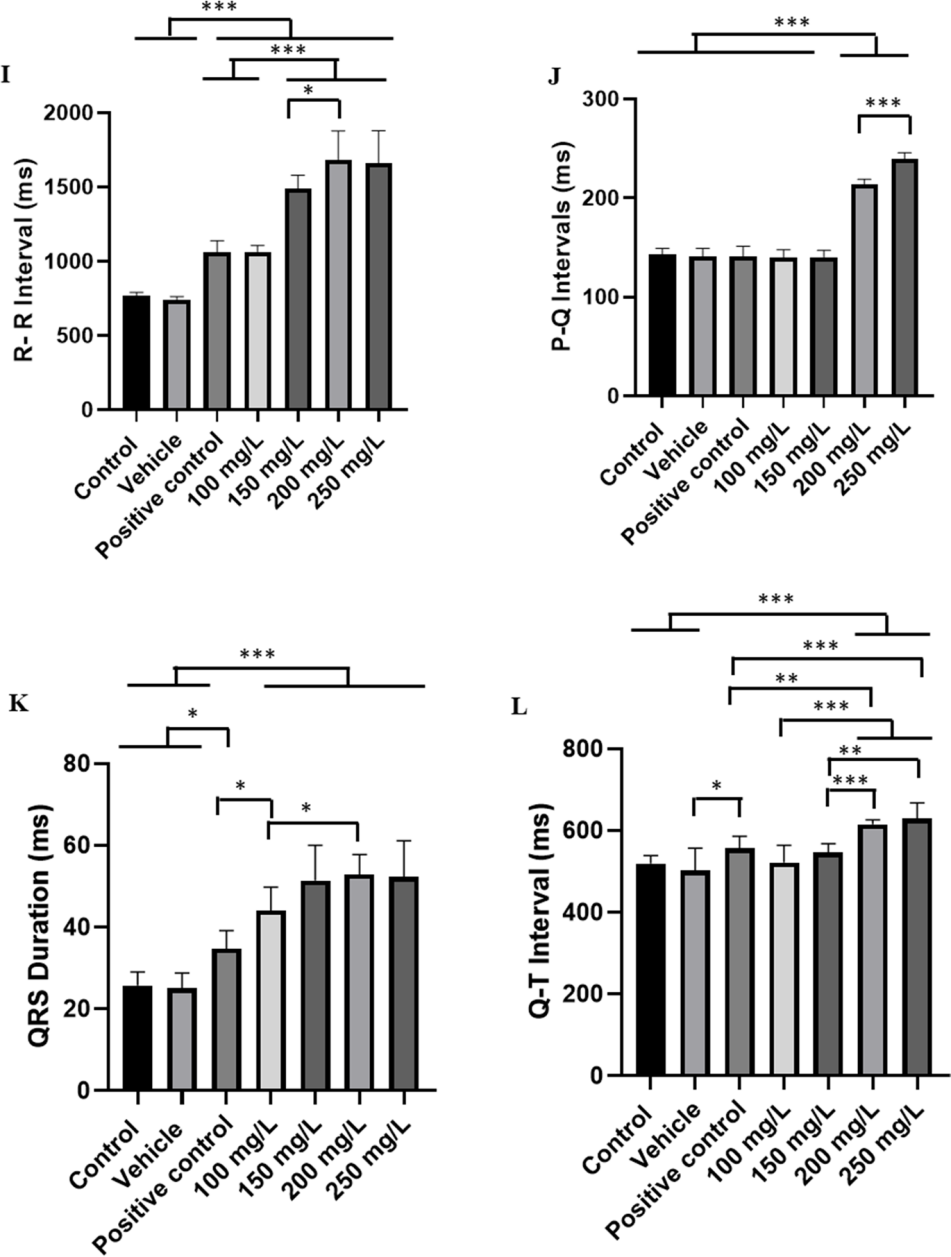
Cardiac activity recording in juvenile tilapia during immersion bath with menthol (left), amplification of the recording in the last 10 s (590–600 s), showing cardiac activity (right). For the following groups: **A** vehicle group; **B** positive control group; **C** group treated with menthol 100 mg/L; **D** 150 mg/L; **E** 200 mg/L; and **F** 250 mg/L. Graphs show the mean values for the following graph elements: **G** heart rate (bpm); **H** QRS complex amplitude (mV); **I** mean values of R-R intervals (ms); **J** P-Q intervals (ms); **K** QRS complex duration (ms); and **L** QT interval (ms) in tilapia during immersion in menthol (ANOVA followed by the Tukey test; *p < 0.05, **p < 0.01 and ***p < 0.001; *n* = 9)

For heart rate, the control group was like the vehicle group (*p* = 0.747). During exposure to menthol at the concentrations used, the ECG showed a decrease in cardiac activity, characterized as sinus bradycardia (Fig. 4A-F), with the mean heart rate of the control group being 78.44 ± 2.6 bpm, higher than that of the other groups. The group exposed to 100 mg/L had a mean of 56.44 ± 2.18 bpm, 26.59% lower than the control, but like the positive control group (*p* = 0.999). The group exposed to 150 mg/L showed a decrease of 44.50% compared to the control group. Fish exposed to 200 mg/L showed a reduction of 52.60% and at the concentration of 250 mg/L showed a reduction of 52.30% compared to controls during induction (Fig. 4G). At the concentrations of 200 mg/L and 250 mg/L, heart rate was similarly reduced (*p* = 0.999). The mean amplitude of the QRS complex in the control group was 1.67 ± 0.033 mV, which was similar to the vehicle and positive control groups (*p* = 0.7483), but was greater than the other groups. The group treated with 100 mg/L presented a mean amplitude of 1.23 ± 0.22 mV, which was like the vehicle and positive control groups (*p* = 0.1291). The group treated with 150 mg/L (1.29 ± 0.23 mV); 200 mg/L and 250 mg/L were like the positive control group (*p* = 0.1757) (Fig. 4H).

The mean RR interval of the control group was 769.4 ± 22.03 ms, like the vehicle group (*p* = 0.999), but shorter than the other groups. The group treated with 100 mg/L presented a mean RR interval of 1064 ± 41.32 ms, like the positive control group (*p* = 0.999). The groups treated with 150 mg/L (1488 ± 90.68 mV) and 250 mg/L were similar (*p* = 0.0532). The group treated with 200 mg/L vs 250 mg/L were similar (*p* = 0.996) (Fig. 4I).

The mean PQ interval for the control group was 143.0 ± 6.10 ms, similar to the vehicle (*p* = 0.9917), positive control (*p* = 0.9986), 100 mg/L menthol (*p* = 0.9761), and 150 mg/L (*p* = 0.9869) groups. The 200 mg/L menthol group had a mean of 214.2 ± 4.71 ms, shorter than the 250 mg/L bath group (239.3 ± 6.32 ms). The groups exposed to 200 mg/L and 250 mg/L had longer mean P-Q intervals than the other groups (Fig. 4J).

The mean QRS complex duration for the control group during induction was 25.67 ± 3.39 ms, which was similar to the vehicle group (*p* = 0.999). The groups treated with 100 mg/L (44.11 ± 5.62 ms) (*p* = 0.999) and 150 mg/L were similar (*p* = 0.1617). The groups treated with 200 mg/L (52.89 ± 4.8 ms) and 250 mg/L were similar (*p* = 0.999) (Fig. 4K).

The mean QT interval during induction for the control group was 518.9 ± 19.77 ms and was similar to the vehicle (*p* = 0.9449), positive control (*p* = 0.2389), 100 mg/L (*p* = 0.999), and 150 mg/L (*p* = 0.5754) groups. These groups were shorter than the 200 mg/L (615.1 ± 11.53 ms) and 250 mg/L (629.3 ± 38.4 ms) groups. However, the 200 mg/L and 250 mg/L groups were similar (*p* = 0.9726) (Fig. 4L).

**Fig. 5:**
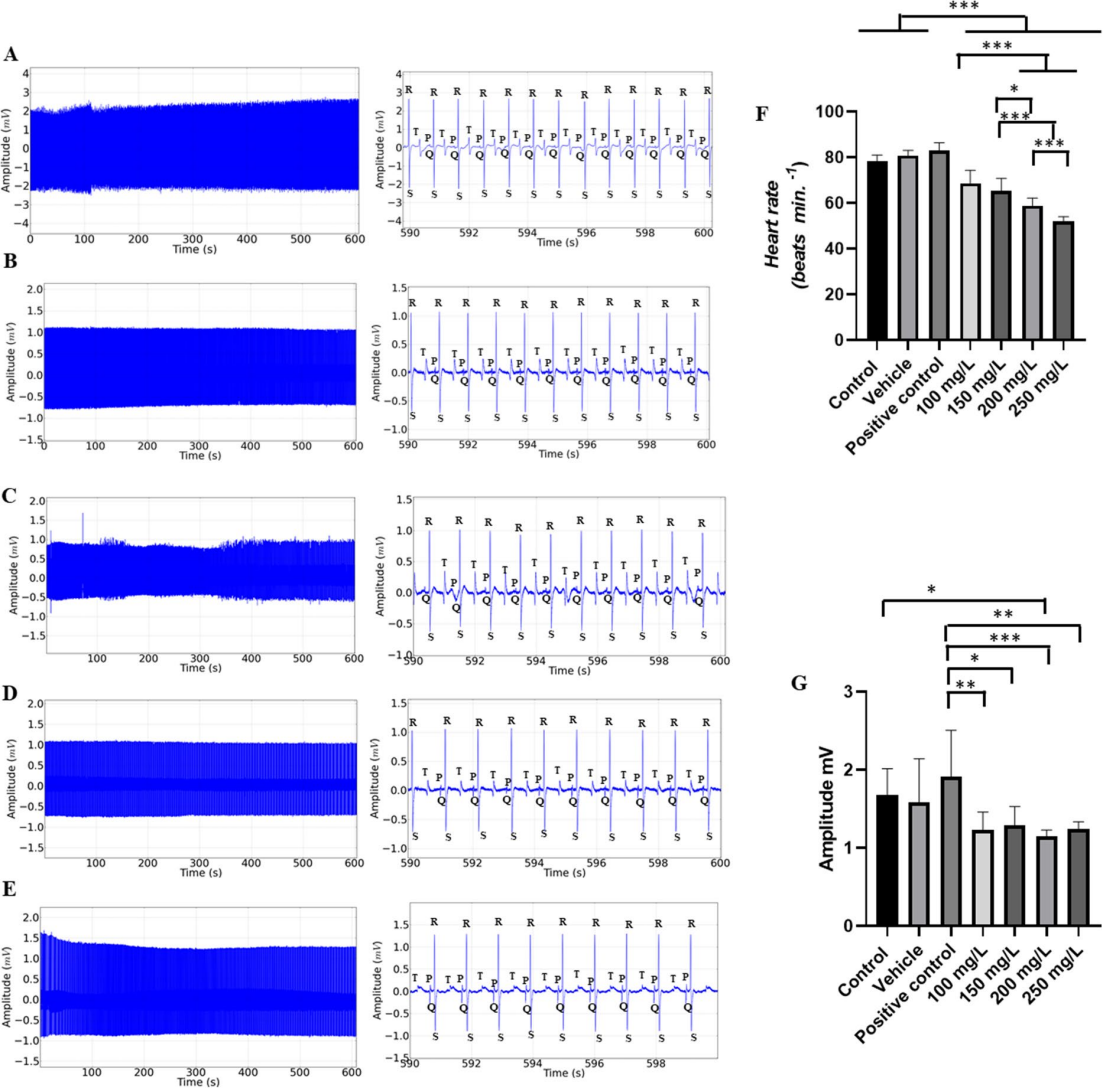

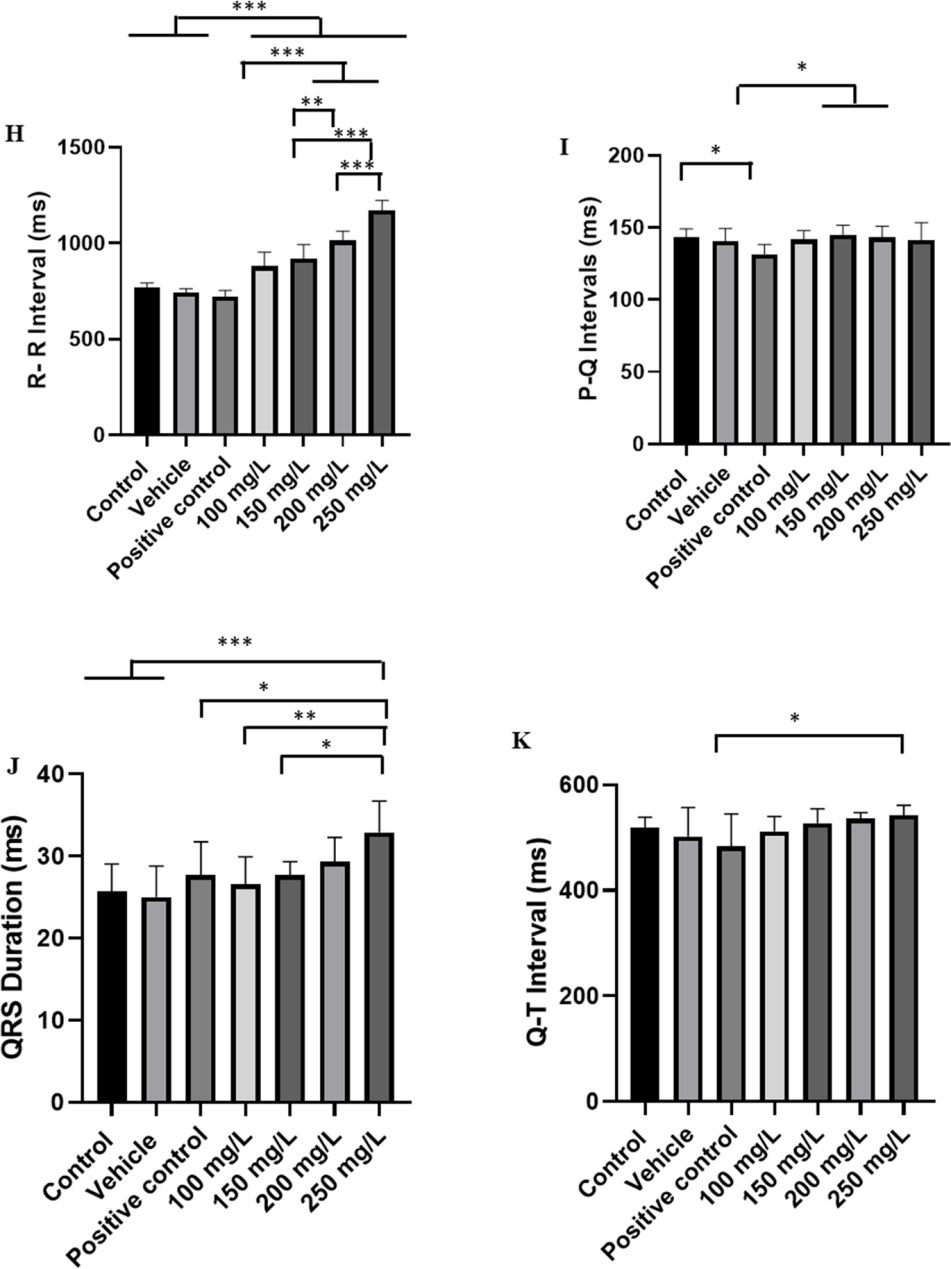
Recording of cardiac activity in juvenile tilapia during recovery after an immersion bath with menthol (left), amplification of the recording lasting 10 s with indication of cardiac graphoelements (center). Evaluated during anesthetic recovery for the following groups: **A** positive control group; **B** after an immersion bath with 100 mg/L of menthol; **C** 150 mg/L; **D** 200 mg/L; **E** 250 mg/L; **F** graph showing the mean heart rate; **G** graphs showing the mean values of the amplitude of the QRS complex (mV); **H** mean values of the R-R intervals (ms); **I** P-Q intervals (ms); **J** duration of the QRS complex (ms); **K** QT interval (ms) (ANOVA followed by Tukey’s test; *p < 0.05, **p < 0.01 and ***p < 0.001; *n* = 9)

During recovery from exposure to menthol concentrations of 100 mg/L, 150 mg/L, 200 mg/L, and 250 mg/L, slow reversibility of sinus bradycardia was observed; thus, the recovery was gradual according to the exposed concentration, the higher the contraction, the slower the recovery of cardiac function (Fig. 5A-E); however, no arrhythmias were observed during recovery, demonstrating that it is a safe drug for the species tested. The heart rate of the control group was 78.44 ± 2.6 bpm, similar to the vehicle and positive control groups (*p* = 0.1472), but higher than the groups treated with menthol. The groups treated with 100 mg/L (68.67 ± 5.74 bpm) and 150 mg/L were similar (*p* = 0.5185). Other treated groups had higher averages (Fig. 5F).

The amplitude of the QRS complex during recovery for the control group was 1.677 ± 0.33 mV, which was similar to the vehicle (*p* = 0.998), positive control (*p* = 0.8108), group treated with 100 mg/L (*p* = 0.1416), 150 mg/L (*p* = 0.2823), and 250 mg/L (*p* = 0.1647) groups. The group exposed to 200 mg/L was similar to the vehicle group (*p* = 0.1603). The group treated with menthol were all similar (*p* = 0.9798) (Fig. 5G).

The mean RR interval during recovery for the control group was 769.4 ± 22.03 ms, which was like the vehicle group (*p* = 0.9124) and positive control (*p* = 0.3838), but was shorter than the other groups treated with menthol. The group treated with 100 mg/L (879.6 ± 73.57 ms) was similar to the group treated with 150 mg/L of menthol (*p* = 0.6083). The group treated with 250 mg/L (1170 ± 52.22 ms) was longer than the other groups (Fig. 5H).

The PQ interval during recovery in the control group was 143.0 ± 6.1 ms and was similar to the vehicle (*p* = 0.9949), 100 mg/L (*p* = 0.999), 150 mg/L (*p* = 0.998), 200 mg/L (*p* = 0.999), and 250 mg/L (*p* = 0.999) groups. The positive control group was similar to the 100 mg/L (*p* = 0.1100) and 250 mg/L (*p* = 0.1175) groups (Fig. 5I).

The duration of the QRS complex in the recovery control group (25.67 ± 3.39 ms) was similar to the vehicle (*p* = 0.999), positive control (*p* = 0.8686) groups, group treated with 100 mg/L of menthol (*p* = 0.997), 150 mg/L (*p* = 0.868), and 200 mg/L (*p* = 0.2611). The group treated with 250 mg/L (47.33 ± 4.0 ms) was similar to the group treated with 200 mg/L (*p* = 0.2952) (Fig. 5J).

During recovery, the QT interval for the control was 518.9 ± 19.77 ms, similar to the vehicle (*p* = 0.9595), positive control (*p* = 0.3942), 100 mg L^−1^ menthol treated group (*p* = 0.999), 150 mg/L (*p* = 0.999), 200 mg/L (*p* = 0.9540), and 250 mg/L (*p* = 0.7953) (Fig. 5K).

**Fig. 6:**
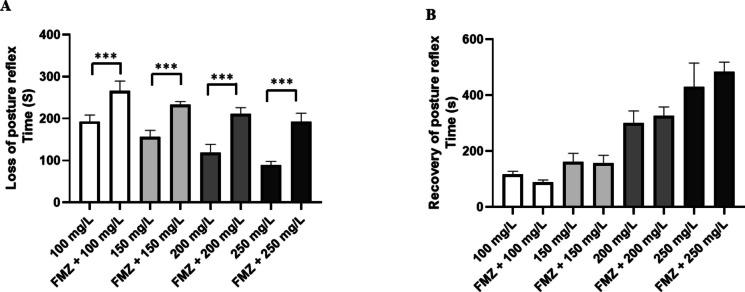
The graphs demonstrate the influence of flumazenil on the latency for anesthetic induction (**A**) and anesthetic recovery by menthol (**B**) (ANOVA followed by Tukey’s test; *p < 0.05, **p < 0.01, and ***p < 0.001; *n* = 9)

After application of flumazenil, the animals submitted to anesthetic induction with menthol presented greater latency. For the group treated with 100 mg/L, the increase in latency was 37.85%; the phenomenon was repeated in the groups treated with 150 mg/L (48.37%), 200 mg/L (79.03%), and 250 mg/L (116.09%), demonstrating mechanisms dependent on the GABA_A_ receptor during induction (Fig. 6A).

Flumazenil did not interfere with the latency for recovery of the postural reflex in the post-anesthesia period; thus, the group treated with 100 mg/L (*p* = 0.8639), 150 mg/L (*p* = 0.999), 200 mg/L (*p* = 0.8760), and 250 mg/L (*p* = 0.1223) were similar (Fig. 6B).

## Discussion

Menthol is a plant extract that, in tilapia, may be able to improve their immunological, physiological, and antioxidant status, being able to protect them even against the toxicity of pesticides present in the environment (Dawood et al. 2020 Among its functionalities, menthol is presented in this study as an anesthetic capable of promoting the loss of the postural reflex within the expected time interval, widely used in very small species such as Danio rerio as well as Nile tilapia, corroborating the data of other authors who presented good results with the use of menthol in tilapia (Ventura et al. 2020; Botrel et al. 2017, Vieira et al. 2025). Navarro et al. (2016) indicate that menthol at a concentration of 75 mg/L was able to induce anesthetic action, control cortisol, and glucose levels in the animals, reducing them even in relation to the control group, in addition to minimizing stress and mortality due to anesthetic toxicity during a transport simulation (Navarro et al. 2016). Concentrations of 200 mg/L and 250 mg/L presented anesthetic induction with lower latencies; however, in the anesthetic recovery period, they presented higher latencies.

The safety of menthol as an anesthetic has already been tested in many studies with other fish species, such as Mazandarani and Hoseini (2017), where menthol was used at a concentration of 512 mg/L in common carp, being recommended for anesthesia and general management (da Cunha et al. 2020); at concentrations of 150 to 250 mg/L, anesthesia was effective and safe for the Guppy species (Zapata-Guerra et al. 2020); menthol was effective for anesthesia in red-bellied pacu fry at a concentration of 50 mg/L. In the case of the study of menthol as an anesthetic for juvenile tambaqui, it was able to promote an effect in lower concentrations of 40 and 60 mg/L using juvenile animals, demonstrating anesthetic safety, with few changes in the ECG (Cantanhêde et al. 2021a, b).

In research carried out with *Piper divaricatum* essential oil in *Colossoma macropomum*, a concentration-dependent relationship was observed, with bradycardia being observed, which intensified at higher concentrations during anesthetic induction (Vilhena et al. 2022). This suggests that the condition of sinus bradycardia may contribute to improving heart contractility (Schwerte et al. 2006). The action of menthol as an anesthetic in Nile tilapia caused a decrease in cardiac activity without the presence of arrhythmias; even at higher concentrations, the sinus rhythm was maintained.

Menthol anesthesia in tilapia is recognized as safe by previous studies, such as those by Marking and Meyer (1985) and Ross & Ross (2009). However, these studies did not use electrophysiological tools and simply evaluated behavior. Our data showed that throughout the recovery period after exposure to menthol concentrations, a gradual reversibility of sinus bradycardia was observed. It is important to emphasize that no arrhythmias were recorded during the recovery process, indicating the safety of this substance in the species subjected to menthol, without significant hemodynamic changes due to heart failure, presenting a reversibility result similar to that of other anesthetics, such as citronella, but with the appearance of arrhythmias during recovery (Barbas et al. 2017). When compared to the positive control (eugenol 64 mg/L), the recovery of cardiac activity was slower with menthol.

An increase in the RR interval was observed in all groups exposed to menthol, and this increase was correlated with a decrease in heart rate during induction. There was an increase in the time interval for each ventricular contraction, which can be observed for other anesthetics (Vilhena et al. 2022). Notably, the effects were more pronounced at higher concentrations of menthol. The positive control had a lesser effect on increasing the RR interval during anesthetic induction. The reduction in heart rate can be attributed to the depressant effect of the anesthetic on the sinoatrial node (de Souza et al. 2019). The highest concentrations of menthol were able to decrease cardiac activity, which may be related to direct actions on cardiac tissue, autonomic actions, or depression of the central nervous system, but without losing the cardiac rhythm with maintenance of sinus rhythm (Cantanhêde et al. 2021a, b). According to Baylie et al. (2010), menthol has antiarrhythmic action by blocking the L-calcium channel in the myocardium, an action like verapamil.

The QRS complex, which corresponds to the contraction of the ventricles, is intrinsically associated with ventricular excitability, as described by Vilhena et al. (2022). Its length provides an indirect assessment of the conduction velocity of the electrical impulse through the ventricular tissue, as established by Cotter and Rodnick (2006). It can be observed that the mean duration of the QRS complex presented significant differences only at the concentration of 250 mg/L during the return period, indicating that at higher concentrations the reversibility of the pharmacological effect of menthol takes longer for anesthetic superficialization, which may indicate a therapeutic window for safe anesthetic procedures.

During induction, there was an increase in the QT interval with the use of menthol in Nile tilapia, in treatments with higher concentrations, which was shown to be reversible during recovery, with no changes that compromise ventricular repolarization, without the presence of arrhythmia, which is advantageous in the use of menthol since this condition would characterize serious arrhythmias on the ECG (Kim et al. 2010; Isbister & Page 2013). The decrease in the PQ interval was observed during anesthetic recovery with the positive control; however, menthol restored the normality of the PQ interval. The PQ interval is an important data because the action of drugs on the heart can cause synaptic blockade, delaying the transmission of impulses through the atrioventricular pathway, according to the study (Vieira et al. 2023).

The bradycardia induced by the anesthetic mechanism of menthol was maintained in sinus rhythm; however, above 200 mg/L, there was an intensification of the bradycardic pattern, which could put the animal at risk. We clearly observed that, based on the concentrations administered, concentrations between 100 and 200 mg/L were the safest regarding hemodynamics, configuring an effective therapeutic window for superficial to deep anesthesia in *Oreochromis niloticus*, with good recovery time. Similar results were reported in tambaqui (*Colossoma macropomum*), in which menthol caused reversible cardiac depression and a significant reduction in heart rate, maintaining sinus rhythm and allowing full recovery after exposure (Cantanhêde et al. 2021a, b). In Danio rerio, menthol also promoted concentration-dependent bradycardia and delayed recovery when administered at high concentrations (Félix et al. 2025). Comparative studies of natural anesthetics corroborate that concentrations above the therapeutic range of 100–200 mg/L tend to prolong recovery and increase cardiovascular depression, characterizing the safe limit of application (da Paz et al. 2024; Bhatt et al. 2023).

According to the study by Watt et al. (2008), testing menthol and propofol to evaluate GABAergic current in vitro; flumazenil did not antagonize the effects of menthol on the GABA_A_ receptor, indicating that the two substances share similar mechanisms of action. According to Szulczyk and Spyrka (2022), menthol has a similar action to carbamazepine in controlling seizures. In addition to its analgesia due to its blockade of calcium channels and voltage-dependent sodium channels (Pan et al. 2012; Dawood et al. 2020), our data show that in vivo, during induction of anesthesia with menthol, flumazenil (1 mg/kg i.p.) interfered by prolonging the time to loss of the animals’ postural reflex. However, there was no change during recovery.

This study demonstrated that menthol acts as an effective anesthetic for *Oreochromis niloticus*, inducing loss of postural reflex with complete recovery, maintaining sinus rhythm throughout the exposure. Concentrations between 100 and 200 mg/L were identified as the ideal therapeutic range, preserving hemodynamic stability and preventing arrhythmias, while higher concentrations such as 250 mg/L intensified bradycardia and delayed recovery, suggesting sublethal cardiovascular toxicity. The observed concentration-dependent reduction in heart rate and QRS amplitude indicates a depressive effect on sinoatrial node activity and myocardial conduction, consistent with previous findings in *Colossoma macropomum* and *Danio rerio*. Moreover, the modulation of anesthetic induction by flumazenil supports the involvement of GABA_A_-mediated pathways in menthol’s mechanism of action. These results highlight menthol’s safety within its therapeutic range but also its potential ecotoxicological relevance, as excessive or environmental exposure could impair cardiac function and physiological homeostasis in aquatic species.

## Data Availability

Data will be made available on request.
